# A Microfluidic Platform with an Embedded Miniaturized Electrochemical Sensor for On-Chip Plasma Extraction Followed by In Situ High-Sensitivity C-Reactive Protein (hs-CRP) Detection

**DOI:** 10.3390/bios12121163

**Published:** 2022-12-13

**Authors:** Zhi-Xuan Lai, Chia-Chien Wu, Nien-Tsu Huang

**Affiliations:** 1 Graduation Institute of Biomedical Electronics and Bioinformatics, National Taiwan University, Taipei 10617, Taiwan; 2 Department of Electrical Engineering, National Taiwan University, Taipei 10617, Taiwan

**Keywords:** microfluidics, whole blood processing, electrochemical (EC) detection, point-of-care (POC) settings, cardiovascular disease (CVD)

## Abstract

Blood testing is a clinical diagnostic tool to evaluate physiological conditions, the immune system response, or the presence of infection from whole blood samples. Although conventional blood testing can provide rich biological information, it usually requires complicated and tedious whole blood processing steps operated by benchtop instruments and well-experienced technicians, limiting its usage in point-of-care (POC) settings. To address the above problems, we propose a microfluidic platform for on-chip plasma extraction directly from whole blood and in situ biomarker detection. Herein, we chose C-reactive protein (CRP) as the target biomarker, which can be used to predict fatal cardiovascular disease (CVD) events such as heart attacks and strokes. To achieve a rapid, undiluted, and high-purity on-chip plasma extraction, we combined two whole blood processing methods: (1) anti-D immunoglobulin-assisted sedimentation, and (2) membrane filtration. To perform in situ CRP detection, we fabricated a three-dimensional (3D) microchannel with an embedded electrochemical (EC) sensor, which has a modular design to attach the blood collector and buffer reservoir with standard Luer connectors. As a proof of concept, we first confirmed that the dual plasma extraction design achieved the same purity level as the standard centrifugation method with smaller sample (100 µL of plasma extracted from 400 µL of whole blood) and time (7 min) requirements. Next, we validated the functionalization protocol of the EC sensor, followed by evaluating the detection of CRP spiked in plasma and whole blood. Our microfluidic platform performed on-chip plasma extraction directly from whole blood and in situ CRP detection at a 0.1–10 μg*/*mL concentration range, covering the CVD risk evaluation level of the high-sensitivity CRP (hs-CRP) test. Based on the above features, we believe that this platform constitutes a flexible way to integrate the processing of complex samples with accurate biomarker detection in a sample-to-answer POC platform, which can be applied in CVD risk monitoring under critical clinical situations.

## 1. Introduction

Blood testing is a clinical diagnostic tool to evaluate physiological conditions, the immune system response, or the presence of infection from whole blood samples [1]. The test includes complete blood cell count (CBC); examining blood cell type, size, and composition [2,3]; monitoring protein, DNA, ion, and metabolite levels [4]; and detecting bacteria [5,6] and viruses [7] in plasma. Due to the complicated whole blood composition, multiple whole blood processing steps—such as plasma extraction, blood cell lysis, reagent mixing, and dilution—are usually required to enable the detection of specific biomarkers [8]. Therefore, conventional blood testing procedures are generally tedious and require well-experienced technicians. Moreover, benchtop instruments and milliliters of whole blood samples, buffer solutions, and reagents are usually needed, limiting the ability to test blood directly in the field, such as in resource-limited or point-of-care (POC) setting environments. To reduce the whole blood sample volume and simplify operational procedures, researchers have developed various microfluidic devices for on-chip whole blood processing and in situ biomarker detection. Among these devices, passive plasma extraction microfluidics are widely applied based on simplified device construction. Without external electrical, optical, or magnetic field assistance, these microfluidics are usually composed of various microstructures—for example, micropillars, microtrenches, microwells, and microfilters—to extract plasma or separate blood cells based on their size, shape, and stiffness [9,10,11]. Briefly, passive plasma extraction microfluidics can be classified into four categories: (1) centrifugation, (2) gravitational sedimentation, (3) hydrodynamic effect, and (4) filtration [8,12]. Among the above methods, filtration is the most time- and size-efficient for plasma extraction with high plasma throughput. However, it usually suffers from hemolysis and clogging issues as the blood cells accumulate on the filter. Although proper blood dilution or an increase in the filtration area could alleviate the above problems, this treatment would also decrease the biomarker concentrations in plasma. Another way to address the hemolysis problem is to reduce the stress acting on blood cells by increasing the membrane porosity [13] or by adding a supporting layer under the membrane [14]. However, the filter membrane fabrication processes are more complicated. The last solution is to combine two passive plasma extraction methods. For example, researchers have placed the filter membrane on the side [15] or the top of the blood sedimentation tube [13,16] to pre-sediment blood cells before plasma extraction through the filtration membrane.

In addition to plasma extraction, another important feature of microfluidics-based blood testing is on-chip biomarker detection. Researchers have developed various label-free sensing mechanisms—such as optical-based surface plasmon resonance (SPR) [17], electrical-based field-effect transistors (FETs) [18], and nanowire sensors [19]—to integrate with microfluidics to perform in situ, continuous, and real-time biomarker detection in blood. However, the above-mentioned methods usually require complicated optical alignments or signal processing to prevent signal interference. Instead, electrochemical (EC) sensors are commonly integrated with microfluidics for in situ biomarker detection based on their simple design, miniaturized reaction volume, and fast response. For example, EC sensors have been used to detect H_2_O_2_ [20,21], glucose [22], cholesterol [23], uric acids, and lactate [24] by enzymatic reactions. Another type of EC sensor is used to detect proteins by functionalizing aptamers or antibodies on the sensor surface. For example, Chikkaveeraiah et al. [25] developed a microfluidic EC immunoassay system to detect prostate-specific antigen (PSA) and interleukin 6 (IL-6). Swensen et al. [26] developed a microfluidic EC aptamer-based sensor (MECAS) for cocaine monitoring. Although the above EC sensors were integrated with microfluidics for the detection of various biomarkers in clinical samples, the microfluidic devices only served as a vehicle for fluid delivery, and multiple off-chip whole blood sample processing steps were still required.

In this study, we developed a microfluidic platform for on-chip plasma extraction and subsequent in situ EC biomarker detection from whole blood samples. Here, we chose C-reactive protein (CRP) as the target biomarker to demonstrate our platform’s features. CRP is an acute-phase protein, and its level increases dramatically in response to inflammation. If the CRP level in blood is higher than 10 μg/mL, the level may indicate acute inflammation resulting from serious infections or other diseases. Another CRP test called high-sensitivity CRP (hs-CRP) test was used to evaluate the risk level of future cardiovascular diseases (CVDs) as low risk (<1 μg/mL), moderate risk (1–3 μg/mL), and high risk (3–10 μg/mL) [27,28]. Therefore, timely CRP monitoring is crucial for predicting potentially lethal CVD events, including heart attacks and strokes, which often occur with no symptoms or signs [29,30,31,32,33,34]. However, the standard CRP or hs-CRP tests in hospitals usually take 5 h and require up to 3 mL of blood samples. To enable a rapid and high-purity plasma extraction, we integrated two plasma extraction features: (1) anti-D immunoglobulin-assisted sedimentation and (2) membrane filtration. To perform in situ CRP detection, we fabricated a three-dimensional (3D) microchannel with an embedded miniaturized EC sensor. As a proof of concept, we confirmed that the dual plasma extraction design achieved the same level of plasma extraction quantity and purity compared with the standard centrifugation method, with smaller sample (100 µL of plasma extracted from 400 µL of whole blood) and time (7 min) requirements. Next, we spiked different CRP concentrations into the whole blood samples to mimic patients with different CVD risk levels and successfully performed on-chip plasma extraction and in situ CRP detection using the embedded EC sensor. The total assay can be performed in 40 min. Based on the above features, we believe that this sample-to-answer platform shows great potential to be applied in CVD risk monitoring under critical clinical situations.

## 2. Materials and Methods

### 2.1. Reagent and Sample Preparation

The self-assembly layer on the EC sensor surface was formed by 11-mercaptoundecanoic acid (11-MUA; 674427) in 99.8% ethanol, activated by a mixture of N-(3-dimethylaminopropyl)-N′-ethylcarbodiimide hydrochloride (EDC; 39391) and N-hydroxysuccinimide (NHS; 130672) in 2-(N-morpholino)ethanesulfonic acid (MES; M3671). The above reagents were all purchased from Sigma-Aldrich (St. Louis, MO, USA). The electrolyte was a 0.1 M, pH 7.4 chloride-free phosphate-buffered saline (PBS) solution containing a mixture of 2 mM potassium ferricyanide (K_3_[Fe(CN)_6_]) and 2 mM potassium ferrocyanide (K_4_[Fe(CN)_6_]·3H_2_O) redox couple (denoted as [Fe(CN)_6_]^3−/4−^). To prevent AuCl formation on the gold electrode surface, PBS was prepared with only sodium dihydrogen phosphate (NaH_2_PO_4_) and sodium hydrogen phosphate (Na_2_HPO_4_), without Cl^−^ ions [35]. Deionized (DI) water (18.3 MΩ cm^−1^) was generated using a Smart2Pure water purification system (Thermo Fisher Scientific, Waltham, MA). Mouse monoclonal anti-human CRP (MCA5880G, clone 160.10G10) and purified human CRP (PHP277) were purchased from Bio-Rad Laboratories (Hercules, CA). Immunoglobulin G (IgG), tumor necrosis factor-α (TNF-α), and bovine serum albumin (BSA) were obtained from Abcam (Cambridge, UK). A volume of 50 μL of Pelikloon anti-D mix (IgG/IgM) monoclonal antibody (K1157, Sanquin, Amsterdam, Netherlands) was coated and vacuum-dried on the bottom surface of the blood container. To preserve efficacy, K_3_[Fe(CN)_6_], K_4_[Fe(CN)_6_]·3H_2_O, NaH_2_PO_4_, Na_2_HPO_4_, 11-MUA, and NHS were stored in a moisture-proof box at room temperature, EDC, TNF-α, IgG and anti-CRP were stored at −20 °C, and MES, BSA, CRP, and anti-D were stored at 4 °C.

### 2.2. Microchannel Fabrication and Assembly

The microchannel was made of two poly(methyl methacrylate) (PMMA) layers (Chi-Mei, Tainan, Taiwan) and machined by a 4-axis computer numerical control (CNC) machine (MODELA MDX-50, Roland, Irvine, CA) using Ø2 and Ø1 mm two-blade end mills. The spindle rotation speed was set at 15,000 rpm. The feed rate was 660 and 420 mm*/*min for the Ø2 and Ø1 mm end mills, respectively. The step length was 0.05 mm. After the machining process, the two PMMA layers were clamped to the EC sensor with a sealing O-ring to assemble the 3D microchannel. The schematic of this assembly step is shown in Figure S1A. Then, on both ends of the 3D microchannel, two micromachined Luer connectors were connected to a 0.22 μm pore size syringe filter (SLGVX13NL, Merck Millipore, Burlington, MA) with the blood container below (plasma extraction section), and to the buffer syringe, respectively.

### 2.3. Instrument Setup

The EC sensor was a thin-film gold electrode chip with interdigitated working electrodes (ED-IDA6-Au, Micrux, Gijón (Asturias, Spain) (Figure S1B). The three electrodes (working, counter, and reference electrodes) were made of 150 nm thin-film gold and a 50 nm Ti layer on a glass substrate. The sensor dimensions were 10 mm, 6 mm, and 0.75 mm in length (L), width (W), and thickness (T), respectively. The interdigitated working electrodes were constructed from 30 pairs of 5 µm wide microelectrodes with 5 µm gaps. Regions outside the Ø2 mm electrochemical detection region and contact panels were protected by an insulating layer made of EPON SU-8 resin. To perform electrochemical impedance spectroscopy (EIS) measurements, the EC sensor was connected to the potentiostat (Autolab PGSTAT204, Metrohm, Herisau, Switzerland), applying a sinusoidal voltage of 0.06 Vrms from 10^−1^ to 10^5^ Hz. A complete EIS scanning measurement took 200 s. After the EIS measurement, the Nyquist plot was analyzed by an electrochemical circle-fitting tool to determine the diameter of the semicircle in the spectrum, representing the charge-transfer resistance (R_ct_) value.

## 3. Results and Discussion

### 3.1. Operation Protocol of the Microfluidic Platform

**Figure 1:**
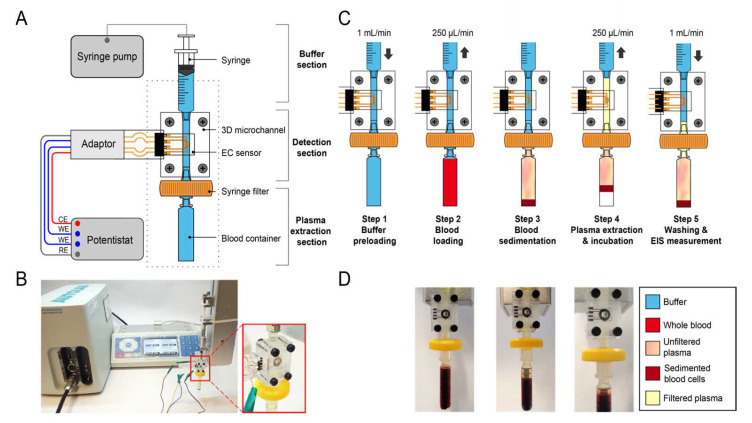
(**A**) The schematic and (**B**) a photo of the microfluidic platform. The red-framed inset shows the composition of the microchannel with an embedded electrochemical (EC) sensor, connecting the blood container and the buffer syringe. (**C**) The operation protocol of the microfluidic platform: (1) buffer preloading; (2) blood loading; (3) blood sedimentation; (4) plasma extraction and incubation; (5) washing and electrochemical impedance spectroscopy (EIS) measurement. (**D**) Photos of blood loading (step 2), sedimentation (step 3), and plasma extraction (step 4) in the microfluidic platform.

The schematic and a photo of the microfluidic platform are shown in Figure 1A,B, respectively. Briefly, the platform is composed of three sections: (1) a plasma extraction section, (2) a detection section, and (3) a buffer section. The plasma extraction section includes a 400 μL tip-shaped blood container for anti-D immunoglobulin-assisted gravitational sedimentation and a syringe filter to block residual blood cells. Anti-D agglutinates with RhD-positive red blood cells (RBCs) (99.7% in the Taiwanese population, 94.6% in the Indian population [36,37]) and increases the erythrocyte sedimentation rate (ESR), facilitating the sedimentation process [38,39]. Notably, the plasma extraction section can also process RhD-negative blood, with a longer sedimentation time. The detection section is a 3D microchannel, where the EC sensor is embedded with a circular detection area of 7 mm^2^. The buffer section contains a storage syringe filled with [Fe(CN)_6_]^3−/4−^, serving as the washing buffer and the electrolyte, operated by a bidirectional syringe pump.

The operation protocol of the microfluidic platform is shown in Figure 1C. Briefly, it can be divided into five steps: First, the microchannel was filled with the electrolyte to exclude any air inside. Second, 400 µL of whole blood was drawn into the blood container from an Eppendorf tube. Third, the whole blood sedimented in the blood collector for 7 min. Fourth, the extracted plasma was passed through the filter membrane into the detection section, followed by 30 min of incubation to allow antibody–antigen interaction. Lastly, the EC sensor surface was washed with the electrolyte, and in situ EIS measurements were performed. The buffer loading and blood drawing flow rates were 1 and 250 µL*/*min, respectively. Photos of the blood loading (step 2), sedimentation (step 3), and plasma extraction (step 4) in the microfluidic platform are shown in Figure 1D.

### 3.2. Plasma Extraction Efficiency

**Figure 2:**
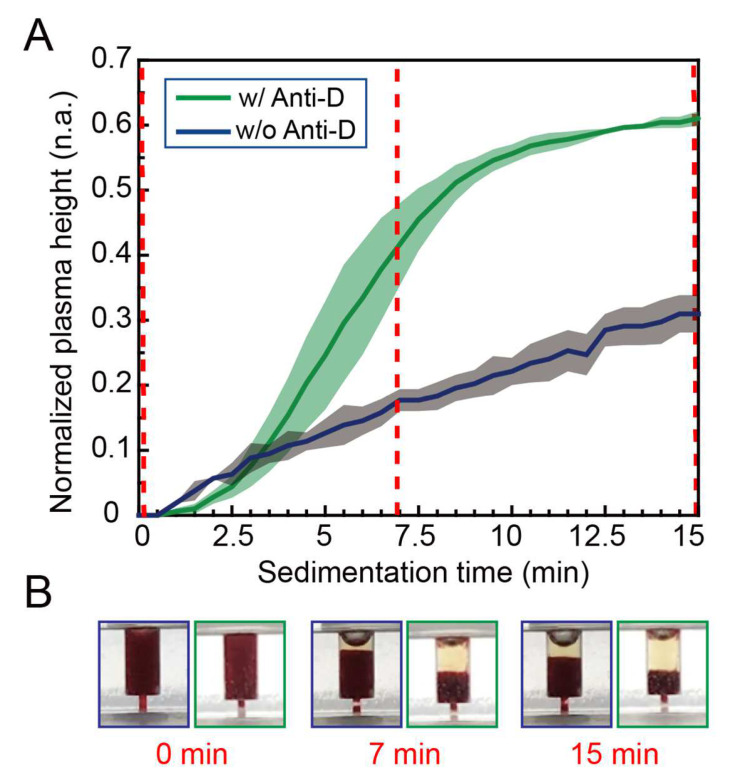
(**A**) The time-lapsed normalized plasma height and (**B**) a series of photos of blood sedimentation in the blood containers with (green line) and without (blue line) anti-D treatment at 0, 7, and 15 min.

To evaluate the sedimentation efficiency of RBCs with anti-D assistance, we preloaded 8 μL of anti-D into a 16 μL blood container (assuming a hematocrit of <50%) and vacuum-dried it for 2 h. The dried anti-D was then coated on the bottom surface of the container. Next, 16 μL of whole blood was loaded into the blood containers with and without anti-D treatment. Then, we observed the erythrocyte sedimentation rate (ESR) by taking a series of photos every 30 s for 15 min and measured the normalized plasma height in the blood container. Figure 2A shows the normalized plasma height (the plasma height/the height of the blood container) with (green line) and without (blue line) anti-D treatment throughout the sedimentation time. Figure 2B shows the blood sedimentation photos in the blood containers treated with (green box) and without (blue box) anti-D at 0, 7, and 15 min. At 7 min, the normalized plasma height of the sedimented whole blood sample in the blood container was > 40%—much higher than that of the sample without anti-D treatment. After 7 min, the sedimentation effect of the anti-D-treated column was less pronounced. Based on the above results, we chose 7 min as the sedimentation time for the subsequent experiments.

**Figure 3:**
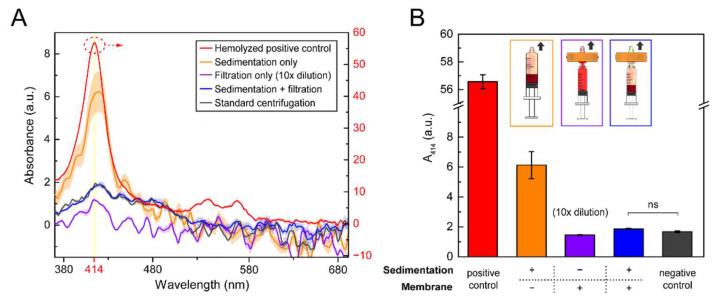
Plasma extraction performance under different whole blood processing methods: (**A**) The UV–Vis absorbance spectra of the extracted plasma processed by (1) artificially hemolyzing whole blood followed by centrifugation (red line; the optical density (OD) value is shown on the right Y-axis), (2) sedimentation only (orange line), (3) filtration only (with 10× dilution; purple line), (4) sedimentation + filtration (blue line), and (5) standard centrifugation (black line). The light bandwidth represents the corresponding standard deviations (*n* = 3). (**B**) The absorbance spectra at 414 nm (A_414_) of the five whole blood processing methods. The inset shows the operation schematics of the sedimentation-only, filtration-only, and sedimentation + filtration cases.

After confirming that anti-D treatment improved the plasma extraction efficiency during the sedimentation process, we then validated the plasma extraction purity of combining sedimentation and filtration by measuring the hemolysis effect, which is a major issue in membrane filtration due to the high flow resistance and fragility of RBCs. Specifically, we measured the sample absorbance at 414 nm (A_414_: the major absorbance peak of hemoglobin) using a spectrophotometer (NanoDrop One, Thermo Fisher Scientific, Waltham, MA, USA). To prepare a positive control sample, we artificially hemolyzed the whole blood and collected the supernatant plasma using centrifugation, indicating a high hemolysis condition (red line in Figure 3A, using the right *Y*-axis). We also prepared a negative control sample using the standard centrifugation process (500 g for 10 min), indicating a low hemolysis condition (black line in Figure 3A). In addition to positive and negative control cases, we prepared (1) sedimentation-only (orange line in Figure 3A), (2) filtration-only (with 10 × dilution; purple line in Figure 3A), and (3) sedimentation + filtration (blue line in Figure 3A) cases for comparison. The absorbance spectra of the above five cases and the corresponding A_414_ intensity are shown in Figure 3A,B, respectively. As shown in Figure 3B, the A_414_ of the hemolyzed positive control sample (red box in Figure 3B) was up to optical density (OD) 57, which is almost 10 times higher than the second largest value, indicating abundant hemoglobin. The A_414_ of the sedimentation-only case (orange box in Figure 3B) was recorded as OD 6.1, with the largest variation, indicating that the sedimentation-only method was less robust. For the filtration-only case (purple box in Figure 3B), the filtration process experienced serious clogging and required up to 10× dilution to collect enough plasma (~30 μL). The A_414_ of the diluted plasma was found to be OD 1.5 (purple box in Figure 3A). In contrast, without dilution, the A_414_ of the sedimentation + filtration case (blue box in Figure 3B) was OD 1.8-as low as the standard centrifugation case of OD 1.7 (black box in Figure 3B). The statistical *p* value was above 0.05, indicating no significant difference. With the least hemoglobin observed, this A_414_ value was 0.3-fold and 0.1-fold lower than that of the sedimentation-only and filtration-only cases, respectively. Specifically, the sedimentation + filtration method produced plasma with purity as high as that of standard centrifugation. We believe that the sedimentation pretreatment minimized the number of trapped RBCs on the membrane and, thus, prevented hemolysis during filtration. Based on the above results, we can conclude that by combining the anti-D immunoglobulin-assisted sedimentation and membrane filtration, the purity of the extracted plasma can be significantly improved.

### 3.3. The Electrochemical (EC) Sensor Functionalization Protocol

**Figure 4:**
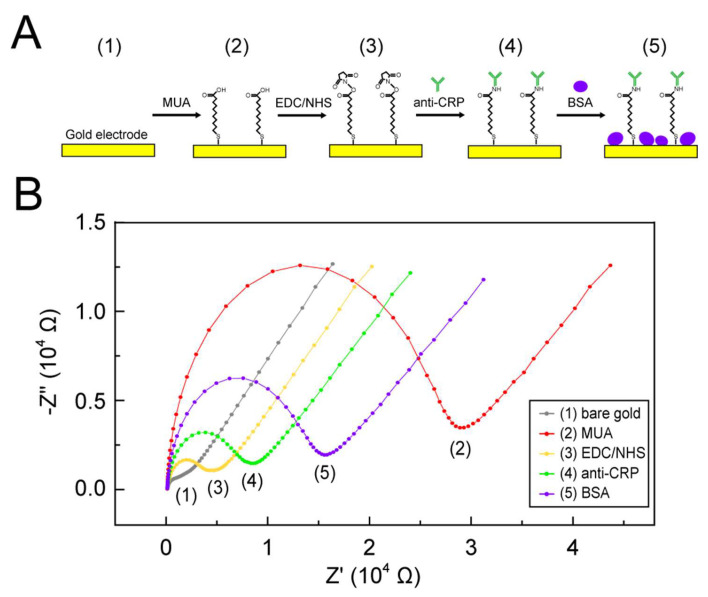
(**A**) The schematic of the EC sensor functionalization protocol. It can be divided into five steps: (1) surface cleaning, (2) 11-MUA self-assembly, (3) EDC/NHS activation, (4) anti-CRP binding, and (5) BSA blocking step. (**B**) The Nyquist plots of EIS measurement at each functionalization step.

Next, we validated the EC sensor functionalization protocol by performing EIS measurements and analyzing R_ct_ changes. The schematic of the modification protocol and the Nyquist plots of the EIS results at each step are shown in Figure 4A,B, respectively. First, the EC sensor surface was cleaned by rinsing with DI water and ethanol several times, followed by continuous CV sweeping between −1 and +1 V at a scan rate of 0.05 V/s with a 0.01 V step, until the spectra overlapped. After cleaning, the R_ct_ value (i.e., the diameter of the semicircle) of the bare surface was around 1000 Ω (gray line in Figure 4B). Such a small value indicates a clean gold surface with an extremely low surface barrier for electron transport. Second, the EC sensor was immersed in 10 mM 11-MUA overnight at 4 °C to construct a stable self-assembled monolayer (SAM) through strong gold–sulfur bonds formed by spontaneous reactions between gold and thiol. After MUA coating, the R_ct_ value increased up to 20-fold (red line in Figure 4B). This large interface resistance can result from electrostatic repulsion between carboxylate groups (–COO^–^) and [Fe(CN)_6_]^3−/4−^ ions, hindering the approach of the redox species to the electrode surface and thus suppressing electron transfer across the interface [40,41,42,43]. The carboxyl group (–COOH)-terminal SAM causing the largest R_ct_ through the functionalization was also observed in previous studies [40,41,44], suggesting successful MUA coating in our study. Third, the EC sensor was incubated in a mixture of 0.1 M EDC and 0.05 M NHS in 0.1 M MES for 40 min at room temperature to activate the carboxyl functional group (–COOH) of MUA via the two-step coupling of EDC and NHS, forming an NHS ester for subsequent antibody binding. After activation, the R_ct_ value was significantly decreased down to 0.2-fold (yellow line in Figure 4B). This decrement arose from the neutralization of the SAM’s terminal groups, promoting the transport of negatively charged redox species to the electrode surface for electron transfer [41,45]. The R_ct_ decrement after EDC/NHS activation was also consistent with the findings of other studies [40,41,43,44,45,46]. Fourth, the EC sensor was treated with 100 µg*/*mL anti-CRP for 30 min at 4 °C to immobilize the anti-CRP through amide bonds formed by the reaction between the NHS ester of the activated surface and primary amines of the anti-CRP, where the NHS served as the leaving group. After anti-CRP immobilization, the R_ct_ values doubled (green line in Figure 4B) since the anti-CRP layer acts as a barrier to the penetration of redox species and thus obstructs electron transfer. Finally, the EC sensor was immersed in 1% BSA for 30 min to block the remaining surface to prevent non-specific binding. The R_ct_ value almost further doubled (purple line in Figure 4B) because of the increased interface barrier of BSA attachment. In brief, the serial R_ct_ changes across the five steps confirmed a successful functionalization.

### 3.4. Specificity Test

Due to the complex composition of whole blood, it is important to ensure a high molecular binding specificity. Here, we used BSA blocking to prevent non-specific binding on the electrodes. To ensure the effectiveness of the blocking, we choose two plasma proteins—immunoglobulin G (IgG) and tumor necrosis factor-α (TNF-α)—to test the specificity of the EC sensor. IgG is the most abundant type of antibody in the blood, constituting 75% of human serum antibodies. Moreover, the molecular weight of IgG (150 kDa) is similar to that of pentameric CRP (115 kDa). On the other hand, TNF-α (17 kDa) represented the non-specific binding behavior of small proteins. In the specificity test, the EC sensor was first incubated with PBS for 30 min to determine the blank R_ct_ value (R_ct, blank_). Then, the EC sensor was sequentially treated with 100 ng*/*mL TNF-α, IgG, and CRP for 30 min, with EIS measurements after each incubation (Figure S2A). The R_ct_ value of CRP was significantly larger than that of the other two non-specific binding molecules (by up to 30%). To further quantify the signal difference, the ΔR_ct_ value (ΔR_ct_ = R_ct, sample_ – R_ct, blank_) was plotted, as shown in Figure S2B. The ΔR_ct_ values of TNF-α and IgG were statistically the same as that of the blank (PBS), while CRP exhibited significant electrochemical signals, indicating that negligible non-specific binding events occurred on the sensor’s surface. Thus, the successful surface blocking with BSA was validated.

### 3.5. CRP Detection

**Figure 5:**
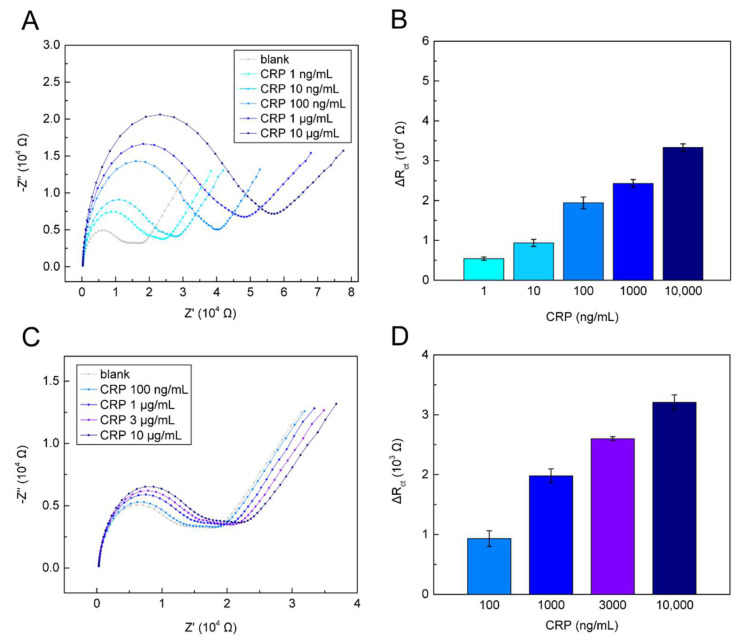
C-reactive protein (CRP) detection performance of the microfluidic platform: The Nyquist plots of EIS measurements and the calculated measured CRP concentrations (*n* = 3) in the detection of CRP spiked in (**A**,**B**) plasma and (**C**,**D**) whole blood samples.

To verify the EC sensor’s performance, we first detected CRP spiked in pre-purified plasma. Here, the plasma was extracted from whole blood using standard centrifugation at 500 g for 10 min. The clinical whole blood samples were collected using a blood collection tube coated with ethylenediaminetetraacetic acid (EDTA) (BD Vacutainer^®^ spray-coated with K_2_EDTA). All clinical experiments were performed following the relevant guidelines and regulations. The experiments were finished within 24 h after sample collection. The CRP samples at 1 ng/mL–10 μg/mL were directly spiked in the plasma, loaded into the microchannel, and incubated with the embedded EC sensor for 30 min. Then, [Fe(CN)_6_]^3−/4−^, as the washing buffer and the electrolyte, flowed downward via a pump to wash out any unbound molecules on the sensor surface, followed by EIS measurements. As shown in Figure 5A,B, the EIS spectra and the ΔR_ct_ value increased along with the spiked CRP concentrations. Finally, we demonstrated the on-chip plasma extraction and in situ CRP detection of our microfluidic platform by measuring spiked CRP in the whole blood samples. For the real CRP diagnostic scenario, we prepared four CRP concentration levels—three CVD risk level thresholds (1000, 3000, and 10,000 ng/mL), and an extremely low level (100 ng/mL)—by spiking CRP into 400 μL whole blood samples. The samples were loaded into the anti-D immunoglobulin-treated blood container of the microfluidic platform at a rate of 250 μL/min, followed by 7 min of sedimentation. Then, 100 μL of supernatant plasma was withdrawn upward at 250 μL/min and filtered by the membrane to remove residual blood cells. Filtered plasma flowed to the EC sensor inside the 3D microchannel for 30 min of incubation. Next, the EC sensor was washed with [Fe(CN)_6_]^3−/4−^, and EIS measurements were performed. The detection results are shown in Figure 5C,D. In the Nyquist plot, clear semicircles and long tails were observed. The ΔR_ct_ values of 100, 1000, 3000, and 10,000 ng/mL CRP were 929, 1981, 2601, and 3209 Ω, respectively. Although the sensitivity was reduced due to the complex molecular interference in the whole blood samples, we still observed significant differences in ΔR_ct_ between 100 ng/mL (low risk), 1000–3000 ng/mL (moderate risk), and 3000–10,000 ng/mL (high risk), which can be applied for the early-stage evaluation of CVD risk levels.

## 4. Conclusions

We proposed a microfluidic platform for on-chip whole blood processing and subsequent in situ CRP detection with an embedded miniaturized EC sensor. Overall, we demonstrated three main features: First, the plasma extraction section integrates anti-D immunoglobulin-assisted sedimentation and membrane filtration to achieve a rapid, no-dilution, high-purity plasma extraction with minimal hemolysis and clogging issues. A volume of 100 μL of plasma was extracted from 400 µL of whole blood within 7 min. Second, the platform successfully detected 1 ng/mL–10 μg/mL CRP spiked in plasma and 0.1–10 μg/mL CRP spiked in whole blood. In clinical whole blood samples, detection down to 0.1 μg/mL CRP was validated, which is below the concentration range of the hs-CRP test (1–10 μg/mL). Lastly, the modular design and the attached miniaturized sensor not only enabled flexible connections but also minimized the assay cost. The embedded universal connectors demonstrated an efficient way to integrate the complex sample processing module with downstream detection. In addition, the detachable design of the 3D microchannel provides the option of serially assembling multiple EC sensors for multiplex biomarker detection. Moreover, due to the miniaturized sensor area (7 mm^2^), the anti-CRP cost is only USD 2 (antibody cost (USD 2) = immobilized antibody concentration (100 μg/mL) X injection volume (30 μL) X antibody stock price (USD 667 per mg)), which is quite cost-effective. Furthermore, the microfluidic channel was made of PMMA, which can be mass-fabricated at low costs. In conclusion, the total assay only took 40 min with 400 µL of whole blood, making it much more efficient than the current methods. Moreover, the platform could detect low CRP concentrations across the range of the hs-CRP test, which has not been demonstrated by commercial POC diagnostic tests such as Abbott AFINION™ CRP cartridges [47]. In the future, our platform can potentially be integrated with commercial blood collection devices such as the Tasso+, which can collect up to 500 μL of whole blood from the arms. Based on the above features, we envision that this sample-to-answer platform could serve as a rapid POC blood testing system for CVD risk monitoring under critical clinical situations.

## Supplementary Materials

The following supporting information can be downloaded at: https://www.mdpi.com/article/10.3390/bios12121163/s1.
