# Supplementary material for: A Microfluidic Platform with an Embedded Miniaturized Electrochemical Sensor for On-Chip Plasma Extraction Followed by In Situ High-Sensitivity C-Reactive Protein (hs-CRP) Detection
Source: Biosensors (Basel). 2022 Dec 13;12(12):1163. doi: 10.3390/bios12121163 (PMC9775575; doi:10.3390/bios12121163)
Supplement: Supplementary file 1 [file biosensors-12-01163-s001.zip › biosensors-2027847-supplementary.pdf]

biosensors

Article

A Microfluidic Platform with an Embedded Miniaturized Electrochemical Sensor for On-Chip Plasma Extraction Followed by In Situ High-Sensitivity C-Reactive Protein (hs-CRP) Detection

Zhi-Xuan Lai [1], Chia-Chien Wu [1] and Nien-Tsu Huang [1,2,*]

[1] Graduation Institute of Biomedical Electronics and Bioinformatics, National Taiwan University, Taipei 10617, Taiwan
[2] Department of Electrical Engineering, National Taiwan University, Taipei 10617, Taiwan
* Correspondence: nthuang@ntu.edu.tw

Electronic supplementary information (ESI)

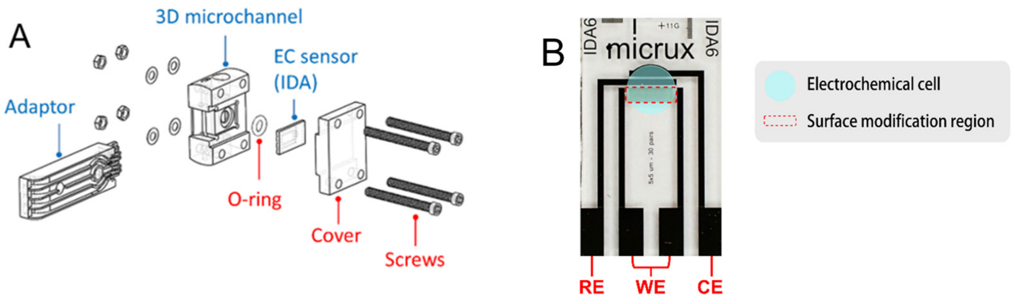

Figure S1. (**A**) An exploded view of the 3D microchannel with an embedded EC sensor. (**B**) A photo of the EC sensor with the three-electrode layout. WE, CE, and RE denote the working, counter, and reference electrodes, respectively.

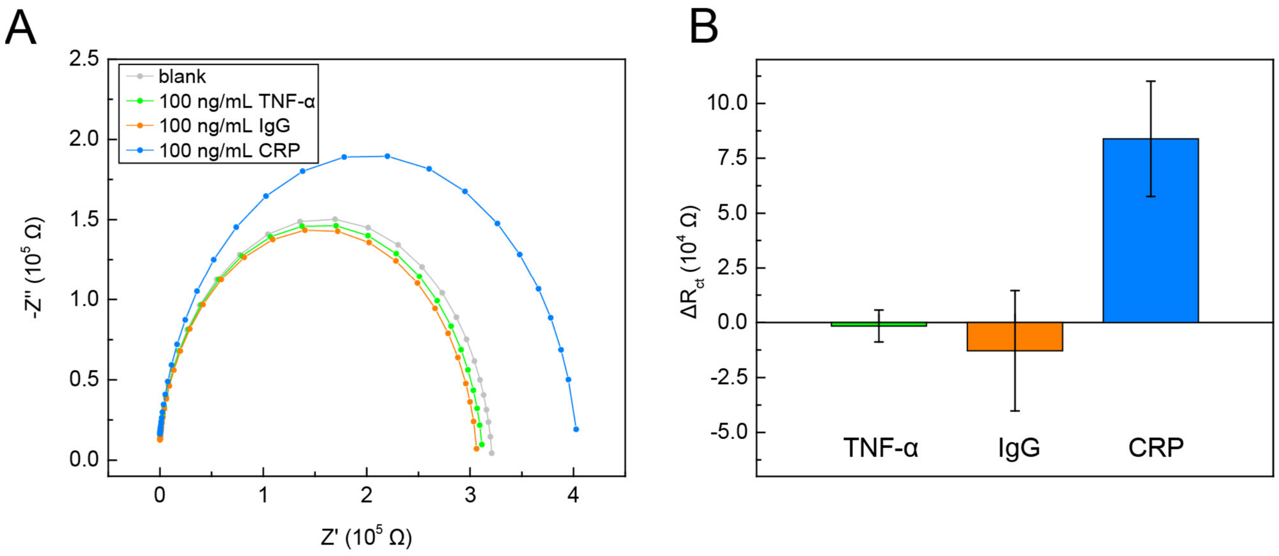

Figure S2. The EC sensor specificity test: (**A**) the Nyquist plots of the EIS measurements, and (**B**) the ΔR_{ct} values (n = 3) of CRP, IgG, and TNF-α.
